# Miniature Broadband NIR Spectrometer Based on FR4 Electromagnetic Scanning Micro-Grating

**DOI:** 10.3390/mi11040393

**Published:** 2020-04-10

**Authors:** Liangkun Huang, Quan Wen, Jian Huang, Fan Yu, Hongjie Lei, Zhiyu Wen

**Affiliations:** 1 Key Laboratory of Fundamental Science of Micro/Nano-Device and System Technology, Chongqing University, Chongqing 400044, China; huangjian7@gmail.com (J.H.); Yu_Fan@cqu.edu.cn (F.Y.); leihongjie@cqu.edu.cn (H.L.); 2 Microsystem Research Center, College of Optoelectronic Engineering, Chongqing University, Chongqing 400044, China; 3 College of Information Engineering, Qujing Normal University, Qujing 655000, China

**Keywords:** micro-NIR spectrometer, scanning grating micromirror, flame-retardant 4 (FR4)

## Abstract

This paper presents a miniaturized, broadband near-infrared (NIR) spectrometer with a flame-retardant 4 (FR4)-based scanning micrograte. A 90° off-axis parabolic mirror and a crossed Czerny–Turner structure were used for creating an astigmatism-free optical system design. The optical system of the spectrometer consists of a 90° off-axis parabolic mirror, an FR4-based scanning micrograte, and a two-color indium gallium arsenide (InGaAs) diode with a crossed Czerny–Turner structure optical design. We used a wide exit slit and an off-axis parabolic mirror with a short focal length to improve the signal-to-noise ratio (SNR) of the full spectrum. We enabled a miniaturized design for the spectrometer by utilizing a novel FR4 micrograte for spectral dispersion and spatial scanning. The spectrometer can detect the full near-infrared spectrum while only using a two-color InGaAs diode, and thus, the grating scanning angle of this spectrometer is small when compared to a dual-detector-based spectrometer. In addition, the angle signal can be obtained through an angle sensor, which is integrated into the scanning micrograte. The real-time angle signal is used to form a closed-loop control over the scanning micrograte and calibrate the spectral signal. Finally, a series of tests was performed. The experimental results showed that the spectrometer has a working wavelength range of 800–2500 nm. The resolution is 10 nm at a wavelength range of 800–1650 nm and 15 nm at a wavelength range of 1650–2500 nm. Similarly, the stability of these two wavelength ranges is better than ±1 nm and ±2 nm, respectively. The spectrometer’s volume is 80 × 75 × 65 mm^3^ and its weight is 0.5 kg. The maximum spectral fluctuation does not exceed 1.5% and the signal-to-noise ratio is 284 after only one instance of averaging.

## 1. Introduction

Near-infrared spectroscopy has a small absorption coefficient and high permeability. It can be used for the direct analysis of solid samples through transmission. The measurement process is relatively fast and pollution-free. Another advantage is that it is easy to maintain, thanks to its simple structure. With the continuous development of MOEMS (Micro-Opto-Electro-Mechanical System) technology [1,2,3], the demand for near-infrared spectrometers in the fields of petrochemicals, food safety, environmental monitoring, biomedicine, and aerospace, among other fields, has dramatically increased [4,5]. Traditional near-infrared spectrometers are generally not only bulky, nonportable, expensive, and consume a lot of power, but they are also hard to use in some conditions. These drawbacks have seriously restricted the application and expansion of near-infrared spectroscopy technology. Thus, cost-effective, high-performance, and wide-spectrum miniature near-infrared spectrometers have been explored for “on-site inspections”.

With the continuous maturation of near-infrared spectroscopy technology, miniature near-infrared spectrometers have been gradually introduced into the market, e.g., Ocean Optics, Avantes, and Hamamatsu Photonics. However, all of these products share a common characteristic: they use fixed diffraction grating and an InGaAs detector array. This configuration inevitably leads to a large product and high production cost. In 2004, F. Zimmer proposed a near-infrared micro-spectrometer that was based on MOEMS scanning grating [6]. MOEMS scanning grating can simultaneously perform spectral dispersion and spatial scanning. It has the advantages of low production costs, a compact size, and low power consumption. The fixed diffraction grating that had been traditionally used was successfully replaced, and a single-tube InGaAs photodiode was used instead of an InGaAs array detector in order to achieve continuous spectrum acquisition, which greatly reduces the overall costs of the spectrometer. As new applications arise, there is a demand for more sophisticated and miniaturized systems. In 2012, Tino Pügner et al. used the latest generation of MEMS (Micro-Electro-Mechanical System) scanning grating to develop a new near-infrared micro-spectrometer with small dimensions and low power consumption [7,8]. The latest generation of MEMS scanning grating devices includes two integrated optical slits and piezoresistive position detection in addition to a miniaturized one-dimensional (1D) scanning grating plate and an electrostatic driving mechanism. A miniaturized scanning grating spectrometer was created for the near-infrared (NIR) range between 950 nm and 1900 nm that had a spectral resolution of 10 nm. In 2016, it was integrated into a mobile phone to meet the demand for mobile applications, as the product was suitable for integration [9,10]. In 2018, Jian Huang also proposed a miniaturized NIR spectrometer that was based on novel MOEMS scanning (tilted) grating [11]. The realization of time-dependent measurements as spectral information was achieved while using an angle sensor integrated into scanning micrograting, with a spectral range of 800–1800 nm and a resolution of 10 nm. However, there are some issues that need be addressed before this type of device can be considered to be a practical alternative. For example, low-frequency and large-aperture MEMS scanning microgrates are usually fragile and they cannot survive shocks due to the brittleness of silicon [12]. Meanwhile, the devices also have high fluctuation when there are environmental vibrations, and the response range of the detector limits the spectral range.

Aiming to broaden the spectral range, Thomas Otto proposed a dual-detector miniature near-infrared spectrometer in 2005 [13], which had a Littrow optical structure design and dual detectors to further broaden the spectral range. As a result, the product achieved a spectral range of 700–1700 nm. After further development, Thomas Otto used this structure to propose a miniature near-infrared spectrometer with a spectral range of 1000–2100 nm, which further broadened the spectral range [14]. However, the spectral ranges of these two spectrometers do not cover the entire near-infrared band, and the Littrow optical structure, which uses a dual optical path system, increases the stray light in the system. Additionally, the spectrometer does not integrate the diffraction grating into the scanning micromirror, which results in an increased volume. The irSys^®^ E NIR series spectrometer, which is based on a dual detector, was developed by the German Fraunhofer Institute for Electronic Nano Systems and produced by the German company TQ-Systems GmbH (gesellschdft mit beschränkter haftung) in 2012 [15]. The wavelength range of this type of spectrometer can be measured in any combination, according to the user’s needs. The spectrum scanning speed is fast and it is extremely lightweight. However, this series of spectrometers also does not integrate the diffraction grating into the scanning micromirror.

We propose a miniature broadband near-infrared spectrometer based on flame-retardant 4 (FR4) scanning micrograting to solve the above issues. Novel FR4 scanning micrograting was used to overcome the limitations of silicon-based MEMS scanning micrograting [16] (this FR4 scanning grating micromirror was verified on our previously developed miniature NIR spectrometer) [17]. Moreover, we used a single-tube, two-color near-infrared detector for the acquisition of the spectral signal, which further broadens the spectral range and simplifying the design of the optical structure. This detector reduces the scanning angle of the dual-detector-based spectrometer. Finally, an off-axis parabolic mirror with a short-focal length was used as a collimating mirror for ensuring the resolution and signal-to-noise ratio of the entire spectral band. Combining this with a crossed Czerny–Turner system, a modified astigmatism-free compact Czerny–Turner optical system was designed. The rest of this article is organized, as follows. In Section 2, we introduce the micrograting, the optical design, and the spectral stitching of the spectrometer. Section 3 presents the characterization and results of the spectrometer. A conclusion is provided in Section 4.

## 2. System Design

In the proposed spectrometer, an FR4-based scanning micrograting is used as the core MOEMS component to achieve spectral dispersion and spatial scanning. Additionally, an optimized and modified optical design using a crossed Czerny–Turner structure and an off-axis parabolic mirror was designed and constructed. Spectral stitching, which is used to stitch together the two spectra from the two-color detector and achieve spectral reconstruction, is the third essential component.

### 2.1. Micrograting

**Figure 1:**
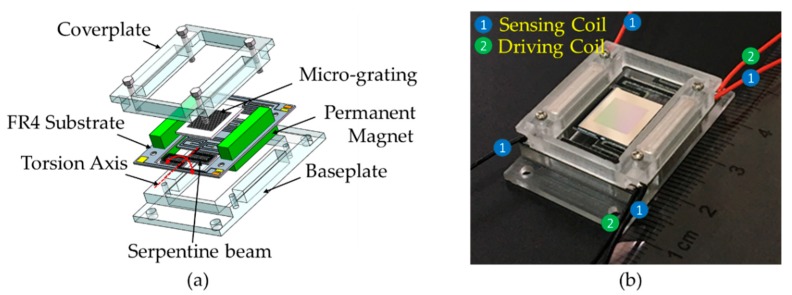
(**a**) Schematic drawing of the assembled flame-retardant 4 (FR4)-based electromagnetic scanning micrograting; and, (**b**) a photograph of the prototype of the FR4 scanning micrograting integrated with a driving coil and a pair of sensors.

The core component used has a scanning dispersive element, which is based on a resonantly driven micromirror that was developed by us that is the key component in the spectrometer. Two kinds of microgratings based on different materials were fabricated in our previous research: single-crystalline silicon [18,19] and FR4 microgratings [20]. In our previous research on FR4 scanning microgratings, it was found that an FR4 scanning micrograting with a serpentine beam structure is suitable for large-aperture and low-frequency MEMS scanning applications [17,21]. We wanted to further broaden the spectral range and improve the imaging quality of the entire spectral range. Therefore, a novel FR4-driven micrograting was selected for spectral dispersion and spatial scanning in an NIR micro-spectrometer: Figure 1a shows a diagram, and Figure 1b shows the prototype of the FR4 scanning micrograting. The corresponding compound control system is also presented [22]. The mechanical half-scan angle of the scanning micrograting reaches 6.3° through the application of a 78 mA current and, when the component is at a resonance frequency of 280.6 Hz, this angle fits within an 800–2500 nm scanning wavelength range. Table 1 lists detailed parameters of the scanning micrograting.

### 2.2. Optical Design

**Figure 2:**
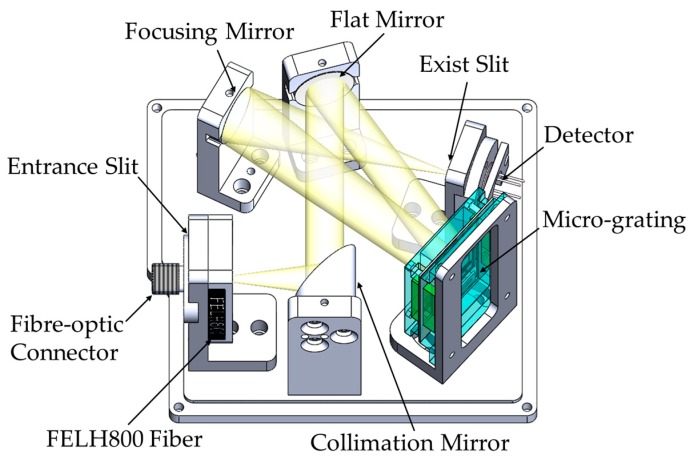
Drawing of the optical layout and assembly of the spectrometer.

Previous experience has illustrated the feasibility of a crossed Czerny–Turner system as part of a modified version [11] in further broadening the spectral range and improving the signal-to-noise ratio. This paper presents a modified crossed Czerny–Turner optical system. First, we used an astigmatism-free compact Czerny–Turner optical design that was based on an off-axis parabolic mirror. Following previous research, the object distance was reduced and the exit slit was enlarged in order to improve the signal-to-noise ratio (SNR) and the dynamic response of the spectrum. Thus, the entrance slit was reduced to improve the resolution. In the design, a single-tube two-color detector reduces the scanning angle of the dual-detector-based spectrometer and it detects the full near-infrared spectrum. Figure 2 shows the optical layout. The light radiation passes through a vertical 40-μm entrance slit aperture and it is collimated by an off-axis parabolic mirror (focal length of 25.4 mm). The parallel beam enters the scanning grating through the plane mirror, and the negative first-order diffraction enters a spherical mirror (focal length of 50 mm), which focuses the beam toward the two-color detector (Hamamatsu K11908-010K, Hamamatsu, Japan) through the vertical 100-μm exit slit aperture. A K1908-010K incorporates an InGaAs PIN photodiode (top InGaAs: cutoff wavelength $\lambda_{c}=1700\mathrm{nm}$) and then mounted over a long-wavelength type InGaAs PIN photodiode (bottom InGaAs: $\lambda_{c}=2550\mathrm{nm}$) along the same optical axis. An 800 nm long-pass filter is used as a second-order filter after the entrance slit, in accordance with the principle of diffraction. 

For a broadband spectrometer, the most straightforward implementation uses spherical mirrors and a plane grating. However, this causes astigmatism, due to the different focal lengths in the tangential and sagittal planes, which result from the off-axis reflections of the spherical mirror. We used an off-axis parabolic mirror instead of a spherical mirror to correct astigmatism and reduce an object’s distance [23]. According to the astigmatism-free Czerny–Turner optical design theory [24,25], astigmatism only exists in the focusing mirror and grating of optical layout components. For a spherical mirror with an incidence angle *θ* and a radius *R*, when a beam is reflected off-axis from a spherical mirror, the tangential focal length and the sagittal focal length become $f_{t}=R\cos\theta /2$ and $f_{s}=R/(\cos\theta /2)$. When combined with Coddington’s equations [26], the tangential and sagittal image distances of the focusing mirror can be expressed as

(1) $$v_{t,s}^{-1}=f_{t,s}^{-1}-u^{-1}$$

where $v_{t}$ and $v_{s}$ are the image distances in the tangential and sagittal plane, respectively; $f_{t}$ and $f_{s}$ are the tangential focal length and the sagittal focal length, respectively; and, *u* is the object distance. The grating acts as a mirror in the sagittal plane, while, in the tangential plane, the grating equation applies, and the image distance in the tangential plane [27] is

(2) $$v_{tg}=-{\left(\frac{\cos\beta }{\cos\alpha }\right)}^{2}\cdot u_{tg}$$

where *α* and *β* are the incident and diffraction angles of the plane grating; and, $v_{tg}$ and $u_{tg}$ are the tangential image distance and the tangential object distance, respectively. The focal length of the off-axis parabolic mirror is half the radius, and the radius of the plane mirror approaches infinity. Otherwise, it is identical, except for the different effective focal lengths of the mirrors. Combining Equations (1) and (2) and sequentially applying the imaging conditions, we obtain

(3) $$S_{s}=\frac{R_{C}R_{F}L_{SC}}{2L_{SC}(R_{C}\cos\theta_{F}-R_{F})+R_{C}R_{F}}$$

(4) $$S_{t}=\frac{R_{C}R_{F}L_{SC}}{2L_{SC}(R_{C}\sec\theta_{F}-R_{F}\frac{\cos^{2}\alpha }{\cos^{2}\beta })+R_{C}R_{F}\frac{\cos^{2}\alpha }{\cos^{2}\beta }}$$

where *L_SC_* is the distance from the entrance slit to the off-axis parabolic mirror; *R_C_* is the radius of the off-axis parabolic mirror; *α* and *β* are the incident and diffraction angles of the plane grating, respectively; and, $\theta_{F}$ and $R_{F}$ are the incident angle and radius of the focusing mirror. If $S_{t}=S_{s}$, the zero-order divergent illumination condition is satisfied. Calculating the value of *L_SC_* by combining Equations (3) and (4) gives the distance *L_FD_* from the focusing mirror to the detector: $L_{FD}=S_{t}=S_{s}$. Finally, all of the parameters are imported into the software ZEMAX and optimized. The off-axis angle *θ_D_* of the detector is given after the final optimization. Table 2 shows the design parameters of the modified optical structure.

**Figure 3:**
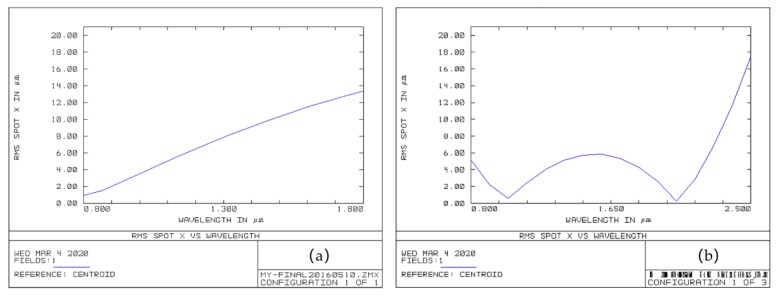
(**a**) The root mean square (RMS) spot *x* of the previous optical structure; (**b**) the RMS spot *x* of the modified crossed Czerny–Turner optical design.

The astigmatism of the optical system can be evaluated by the size of the root mean square (RMS) spot radius *x* (using ZEMAX software, Version, Kirkland, WA, USA) [28]. We compared the modified crossed Czerny–Turner optical design to our previous optical structure to evaluate the broadband astigmatism correction [11], as shown in Figure 3. Obviously, for the previous optical structure, the maximum RMS spot radius *x* was about 14 µm in an 800–1800 nm spectral range, as shown in Figure 3a. However, for the modified crossed Czerny–Turner optical design, the RMS spot radius *x* was approximately 6 µm in an 800–1800 nm spectral range, and the maximum spot was less than 18 µm in the wavelength range from 800 to 2500 nm, as shown in Figure 3b. The results demonstrated that the modified crossed Czerny–Turner optical design corrects astigmatism and it performs better than other models. 

### 2.3. Spectral Stitching

Spectral stitching is an important step for spectrometers that is based on a single-tube, two-color detector. Thanks to the angle sensor coil being integrated into the FR4 scanning micrograting, with our design, the grating position can be monitored in real time. Therefore, the angle sensor signal is used for online real-time spectral stitching. When the micrograting is driven and controlled with a sinusoidal signal, the scan angle of the micrograting over time can be expressed, as

(5) $$\theta (t)=\theta_{max}\cdot \sin(2\pi ft)$$

where *θ_max_* and *f* are the maximum scan amplitude and the resonant frequency, respectively. During the sinusoidal movement of the micrograting, the electromotive force that is generated by the angle sensing coil is

(6) $$U(t)=\left(B\cdot \sum_{j=1}^{n}S_{j}\right)\cdot \theta^{\prime }(t)=\left(B\cdot \sum_{j=1}^{n}S_{j}\right)\cdot 2\pi f\theta_{max}\cos(2\pi ft)$$

where B is the magnetic flux density and $\sum_{j=1}^{n}S_{j}$ is the area sum of all the angle sensing coils. From Equation (6), it can be seen that there is a 90° phase difference between the electromotive force and the scan angle of the grating. This also shows that the initial position of the grating is perpendicular to the neutral plane of the magnetic field. The wavelength detected at this position is defined as the center wavelength. Subsequently, Equation (5) can be substituted into Equation (6):

(7) $$U(t)=\left(2\pi fB\cdot \sum_{j=1}^{n}S_{j}\right)\cdot \theta (t-\frac{1}{4f})$$

**Figure 4:**
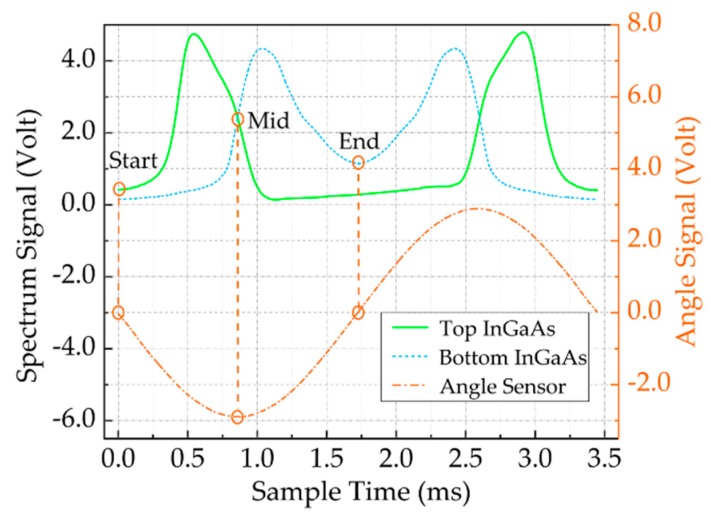
Two full scans within one mirror oscillation and the beginning of the spectrum of the top InGaAs (i.e., 800–1650 nm) and the end of the spectrum of the bottom InGaAs (i.e., 1650–2500 nm), which both correspond to the position where the angle signal output is 0. Their intersection points correspond to the position where the angle signal output is the largest.

Combined with the grating equation, the detected wavelength λ(t) in the time domain can be obtained while using

(8) $$\lambda (t)=d\cdot (\sin(\beta_{mid}+\theta (t))+\sin(\alpha_{mid}+\theta (t)))$$

where $\alpha_{mid}$ and $\beta_{mid}$ represent the incidence angle and diffraction angle of the grating at the center of the wavelength, respectively; and, d is the grating constant. Finally, the spectral signal and angle sensor signal output in the time domain (by the single-tube, two-color detector) can be obtained through the acquisition circuit. The two-color detector, which is different from a single-tube detector in terms of spectral reconstruction, is shown in Figure 4. The top InGaAs and bottom InGaAs acquire two spectra, 800–1650 nm and 1650–2500 nm, respectively. When the grating is scanned from the initial position to the position of the maximum amplitude, the wavelength slides over the detector from the center wavelength to the minimum wavelength or maximum wavelength, so that a spectrum is obtained from 1650 nm to 800 nm or 2500 nm. The same spectrum is obtained when the grating rotates back to the initial position in the opposite direction. Therefore, the start and end points of one spectral period are exactly the two adjacent minimum amplitudes values positions of the angle sensor output, which correspond to the two adjacent maximum amplitudes values positions of the grating plate. The middle point of the spectral period exactly correspond to the maximum amplitude value position of the angle sensor output, where the position of the grating is perpendicular to the neutral plane of the magnetic field.

**Figure 5:**
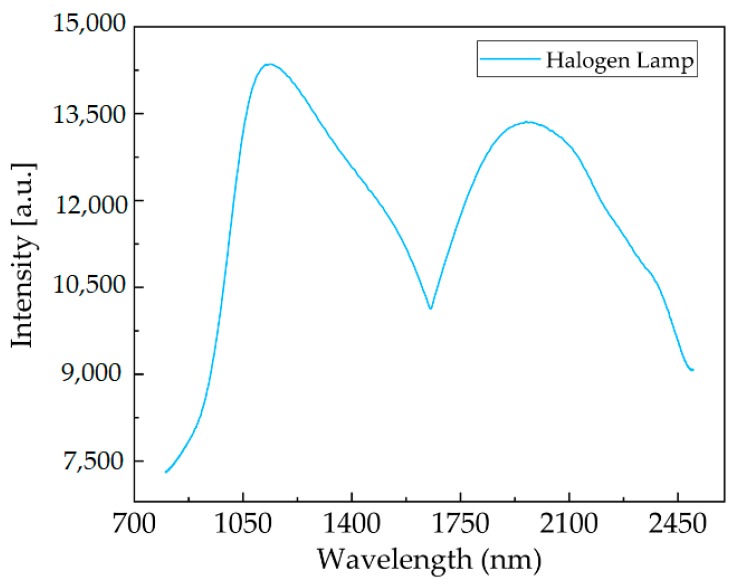
The correlated spectrum stitched from the wavelength of the halogen lamp.

In order to create the stitching spectrum, first, 1650 nm was selected as the center wavelength. The corresponding long- and short-wavelength amplitudes were found using the center wavelength as a reference. Second, adjusting the amplification of the acquisition circuit to make its amplitude equal to the amplitude of the reference spectrum at 1650 nm and prevent breakpoints of the two spectra at the center wavelength during spectrum stitching. Finally, utilizing the relationship between the angle sensor signal U(t) and the wavelength λ(t), the final spectrum I(λ) can be calculated, as shown in Figure 5.

## 3. Characterization and Results

The prototype of the modified spectrometer was successfully assembled and an experimental platform was established. Subsequently, the performance of the prototype was tested as follow.

### 3.1. Wavelength Range

**Figure 6:**
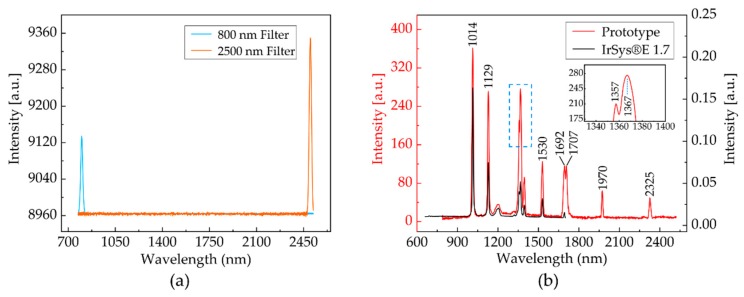
(**a**) Wavelength range of the prototype; and, (**b**) An array-detection-based near-infrared spectrometer and the prototype were tested using a mercury lamp experiment and the illustration indicates the highest resolution of the prototype.

Figure 6b shows the spectral curves after stitching and calibration: a spectral range of 800–2500 nm was acquired when the scanning angle of the grating was 6.3°. Figure 6a shows the measured intensity signal, where two 800 nm and 2500 nm narrowband filters were separately applied in a halogen lamp. The prototype could cover a spectral range from 800 nm to 2500 nm, as the figure indicates. Furthermore, a full spectrum was obtained within less than 1.8 ms, since the FR4 scanning micrograting was operated at 280.6 Hz. 

### 3.2. Spectral Resolution

The spectral resolution of the spectrometer primarily depended on the widths of the input and exit slits and on the reciprocal dispersion of the diffraction grating. Section 2 covers the parameters of the design. The spectral resolution was calculated from the two adjacent peaks of the characteristic spectral lines of the high-pressure mercury lamp light source with a 400-µm silica fiber as the connector between the light source and the spectrometer. An irSys^®^ E 1.7 NIR spectrometer produced by the German company TQ-Systems GmbH was used for a comparative test to verify the accuracy of the test using the high-pressure mercury lamp [15]. The irSys^®^ E 1.7 NIR spectrometer has a wavelength range of 660–1730 nm and resolution of 8 nm. It is worth noting that the irSys^®^ E series of NIR spectrometers includes a long-pass filter within the products. Figure 6b depicts the results of the measurements. As the measurement results suggest, all of the characteristic peaks of the two mercury lamp spectral lines basically coincided [29]; and, two adjacent peaks at 1357 nm and 1367 nm can be clearly distinguished within the spectral range from 800–1650 nm. Similarly, two adjacent peaks at 1692 nm and 1707 nm can also be clearly distinguished in the spectral range from 1650–2500 nm. Thus, this indicated that the resolution in our spectrometer was better than 10 nm for the top InGaAs detector and better than 15 nm for the bottom InGaAs detector [30].

### 3.3. System Stability and Signal-To-Noise Ratio

**Figure 7:**
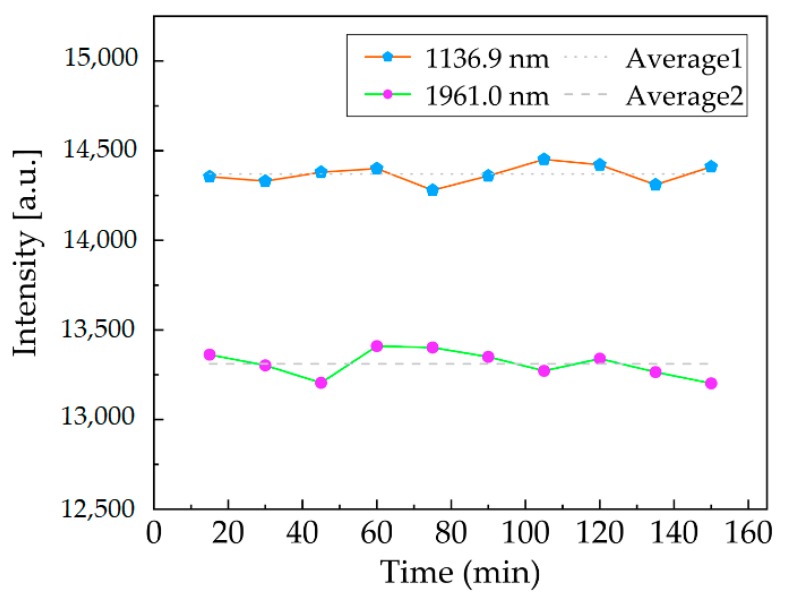
Ten measurements of spectral peak fluctuation at 1136.9 nm and 1961.0 nm.

A calibrated spectrum curve was processed using our laboratory’s spectrum software to obtain the spectral peaks corresponding to two non-cooled detectors and the wavelengths corresponding to these peaks to measure long-term stability. The detection wavelengths of the top (i.e., 800–1650 nm) and bottom (i.e., 1650–2500 nm) detectors were 1136.9 nm and 1961.0 nm, respectively. For a period of 150 min, a set of scanning spectra was acquired every 15 min at ambient temperatures of about 25 °C. We used the 10 measurements to analyze the degree of dispersion of the spectral peaks, as shown in Figure 7. We calculated that the maximum spectral fluctuation did not exceed 1.5%, and the wavelength stability was better than ±1 nm and ±2 nm at 1136.9 nm and 1961.0 nm, respectively. We utilized the standard deviation within a dark spectrum to measure the noise, and the spectral signal was characterized using the average value of the spectrum to calculate the signal-to-noise ratio (SNR). The preliminary measurements suggested that at the said resolution, the SNR was 284 at 1136.9 nm and 188 at 1961.0 nm after only instance of averaging when a single scan acquired within 1.8 ms, respectively. SNRs can be improved by increasing the number of co-added scans since intra-spectral noise can be improved by averaging, but at the expense of measurement time [31,32]. Here, the SNR of full spectrum reached 3928 with 100 added scans. 

We processed and assembled the prototype using the design parameters listed in Table 2. The spectral response covered from 800 nm to 2500 mm (a wide range), and the prototype produces little noise. The spectrometer has a size of 80 × 75 × 65 mm^3^, a weight of approximately 0.5 kg, and it is made of aluminum. Table 3 lists comparisons between the performances of the SGS 1900 [31], the irSys^®^ E 2.4 [15], and the spectrometer prototype. The comparisons show that the spectral range of the prototype is wider than that of the single-detector-based spectrometer, and the size is smaller. Further, when compared to the dual-detector-based spectrometer, the scanning speed is faster, and the SNR is higher.

## 4. Conclusions

In summary, a prototype of a miniature broadband near-infrared spectrometer that was based on FR4 electromagnetic scanning micrograting was presented. When compared to a conventional Czerny–Turner spectrometer based on MOEMS scanning grating, it possesses several noteworthy advantages: it reduces the processing difficulties and costs of fabrication of the core component; increases the shock and vibration reliability; and, overcomes the shortcomings of MOEMS-based scanning grating, where the maximum area dimension is limited by the fabrication parameters of the rotating grating. For an optical design, the light throughput in the system was increased while using large aperture grating design, and further, an off-axis parabolic mirror with a short-focal length was used to improve the system’s imaging quality, thereby improving the signal-to-noise ratio of the spectrometer. Meanwhile, in order to broaden the spectral range, a single-tube, two-color detector with a wide spectral response range was used to detect the full near-infrared spectrum and reduce the grating scanning angle of the spectrometer. A series of test results showed that the scanning range of the spectrometer was 800–2500 nm when the mechanical deflection angle reached ±6.3°, and the time that was taken for a single scan was less than 1.8 ms. The spectral resolution was better than 10 nm in the short-wave range (i.e., 800–1650 nm) and better than 15 nm in the long-wave range (i.e., 1650–2500 nm). The SNR up to 284:1 in the entire near-infrared spectrum using modified optical layout. Furthermore, a reduction of detector noise was achieved through reduced detector’s temperature; thus, the use of a cooled detector could further improve the SNR of the spectrometer.

## Figures and Tables

**Table 1:** Parameters of the FR4-based electromagnetic scanning micrograting.

Parameter	Value
Resonance frequency (Hz)	280.6
Mechanical half-scan angle (°)	6.3
Mirror plate (mm^2^)	7 × 7
Quality factor (Q) (a.u.)	80.5
Grating groove density (lines/mm)	250
Blazed angle (°)	9.4

**Table 2:** The final design parameters of the modified optical structure.

Parameter	Value
Wavelength range (nm)	800–2500
Diffraction order (a.u.)	−1
*d* (µm)	4
*R_C_* (mm)	50.8
*R_F_* (mm)	100
*L_SC_* (mm)	25.4
*L_FD_* (mm)	50.29
*α* (°)	6.5
*β* (°)	17.45
*θ_F_* (°)	15.6
*θ_D_* (°)	3.85

**Table 3:** A comparison of the Corresponding parameters of the SGS 1900, the irSys^®^ E 2.4, and the spectrometer prototype.

Parameter	SGS 1900	irSys^®^ E 2.4	Prototype
Spectral range (nm)	1200–1900	910–2390	800–2500
Spectral resolution (nm)	10	11	10 (800–1650) 15 (1650–2500)
Size (mm^3^)	100 × 80 × 75	138 × 89 × 66	80 × 75 × 65
SNR (a.u.)	1700:1	1000:1	3928:1
Weight (g)	750	840	500
Scan time (ms)	4	4	1.8
